# Identifying people with potentially undiagnosed dementia with Lewy bodies using natural language processing

**DOI:** 10.1038/s41514-025-00252-x

**Published:** 2025-07-18

**Authors:** Mohamed Heybe, Lucy Gibson, Annabel C. Price, Rudolf N. Cardinal, John T. O’Brien, Robert Stewart, Christoph Mueller

**Affiliations:** 1 Institute of Psychiatry, Psychology and Neuroscience, King’s College London, London, UK; 2 South London and Maudsley NHS Foundation Trust, London, UK; 3 Department of Psychiatry, School of Clinical Medicine, University of Cambridge, Cambridge Biomedical Campus, Cambridge, UK; 4 Cambridgeshire and Peterborough NHS Foundation Trust, Fulbourn, Cambridge UK

**Keywords:** Dementia, Diagnosis

## Abstract

Natural language processing (NLP) can expand the utility of clinical records data in dementia research. We deployed NLP algorithms to detect core features of dementia with Lewy bodies (DLB) and applied those to a large database of patients diagnosed with dementia in Alzheimer’s disease (AD) or DLB. Of 14,329 patients identified, 4.3% had a diagnosis of DLB and 95.7% of dementia in AD. All core features were significantly commoner in DLB than in dementia in AD, although 18.7% of patients with dementia in AD had two or more DLB core features. In conclusion, NLP applications can identify core features of DLB in routinely collected data. Nearly one in five patients with dementia in AD have two or more DLB core features and potentially qualify for a diagnosis of probable DLB. NLP may be helpful to identify patients who may fulfil criteria for DLB but have not yet been diagnosed.

## Introduction

Electronic health records (EHRs) hold extensive patient data, offering unique opportunities to identify and assemble cohorts that may be challenging to recruit in traditional, prospective studies^1^. This is particularly true for patients with dementia with Lewy bodies (DLB), a condition often underdiagnosed or misdiagnosed as Alzheimer’s disease (AD) in routine clinical settings due to overlapping cognitive and functional symptoms^2^.

DLB is characterized by a distinct set of core features, including visual hallucinations, cognitive fluctuations, parkinsonism, and rapid eye movement (REM) sleep behaviour disorder (RBD), which form the basis of current diagnostic criteria for DLB and differentiate it from AD^3^. However, in clinical practice, these symptoms are often not asked about, overlooked, or not comprehensively enough documented to recognise the presence of the condition, leading to diagnostic delays or misdiagnosis^4^. This gap in accurate diagnosis has substantial implications, as patients with DLB have a worse prognosis than patients with AD, may respond differently to standard dementia treatments, and are at a higher risk of adverse reactions to certain medications, particularly antipsychotics^5,6^.

The South London and Maudsley NHS Foundation Trust Biomedical Research Centre (SLaM BRC) Case Register is a research repository of anonymised, structured, and open-text data derived from electronic health records for mental health and dementia care. It was developed in 2008 and has since expanded to include over 500,000 patients’ records accessed through the Clinical Record Interactive Search (CRIS) application^7^. Natural language processing (NLP) provides a powerful tool for mining and analysing unstructured data within EHRs, enabling the extraction of clinically relevant information such as symptom patterns and disease markers^8,9^. This is especially powerful when using mental health records, which often contain little structured information, but have much key information captured within text descriptions.

The aim of this study was to apply NLP algorithms to detect core symptoms of DLB in a cohort of patients in Southeast London with documented diagnoses of DLB or AD. We chose core symptoms only as their value as diagnostic markers is clearly established and has repeatedly been reconsidered in consensus criteria^3^. We did not consider supportive features as they have less diagnostic specificity and larger overlap between dementia subtypes^10^. We sought to determine the prevalence of core symptoms across both groups, with a focus on identifying cases where patients diagnosed with dementia in AD may present with a symptom profile consistent with DLB. This approach aims to enhance diagnostic practices by providing insights into the presence of possibly undiagnosed DLB cases within the population of patients with dementia in AD and illustrating the utility of NLP in advancing dementia diagnosis.

## Results

### Sample characteristics

Of the 14,329 patients drawn from the CRIS resource as the sample for this analysis, 617 (4.3%) were diagnosed with dementia with Lewy bodies (DLB) and 13,712 (95.7%) with dementia in Alzheimer’s disease (AD). Compared to patients with dementia in AD, those with DLB were generally younger, both at first referral to mental health services (mean age: 76.9 vs. 80.6 years) and at initial dementia diagnosis (mean age: 78.7 vs. 82.0 years). Patients with DLB were less likely to be female (49.0% vs. 64.0% female in AD) and more likely to be married or cohabiting (42.6% vs. 34.8% married/cohabiting in AD) (see Table 1).

**Table 1:** Demographic characteristics and core symptoms in the full cohort, DLB and Alzheimer’s disease

	Full cohort (*n* = 14,329)	DLB (*n* = 617)	AD (*n* = 13,712)	*p*-value
Sociodemographic characteristics				
Age at first referral in years (mean, SD)	80.5 (8.6)	76.9 (10.2)	80.6 (8.5)	<0.001
Age at first dementia diagnosis in years (mean, SD)	81.8 (8.0)	78.7 (9.1)	82.0 (7.9)	<0.001
Female gender (%)	63.4%	49.0%	64.0%	<0.001
Non-White ethnicity (%)	27.8%	26.4%	27.9%	0.434
Married or cohabiting (%)	35.1%	42.6%	34.8%	<0.001
Core symptoms				
Visual hallucinations (%)	19.6%	83.1%	16.7%	<0.001
REM sleep behaviour disorder (%)	10.0%	26.6%	9.3%	<0.001
Fluctuations (%)	32.2%	74.7%	30.3%	<0.001
Parkinsonism (%)	21.5%	62.1%	19.7%	<0.001
Number of core symptoms recorded				<0.001
No core symptom	46.2%	3.9%	48.1%	
1 core symptom	32.3%	13.0%	33.2%	
2 core symptoms	14.6%	30.4%	13.9%	
3 core symptoms	5.7%	38.1%	4.2%	
4 core symptoms	1.2%	14.6%	0.6%	

### Prevalence of DLB core symptoms in patients with DLB and AD

Core symptoms of DLB were significantly more prevalent in patients with DLB compared to those with AD. Notably, 83.1% of DLB patients exhibited visual hallucinations, while only 16.7% of AD patients did. Similarly, fluctuations (74.7%), parkinsonism (62.1%), and RBD (26.6% of patients with DLB) were markedly higher in DLB than in AD. While a larger proportion of patients with AD presented with no or one core symptom, more patients with DLB presented with 2, 3, or 4 core symptoms (see Table 1).

A substantial subset of patients with dementia in AD also exhibited DLB core symptoms. Specifically, 30.3% had fluctuations, 19.7% exhibited parkinsonism, 16.7% had visual hallucinations, and 9.3% had experienced possible RBD. Among the 13,712 patients with dementia in AD, 33.2% (*n* = 4546) had one DLB core symptom recorded, the criteria needed for a diagnosis of possible DLB, and 18.7% (*n* = 2563) had two or more, suggesting that this latter group could potentially meet the criteria for a probable DLB diagnosis.

### Characteristics and symptomatology of those with at least 2 DLB core symptoms

Further analysis compared patients with dementia in AD who had two or more DLB core symptoms (AD2CS group) to those with fewer than two core symptoms, and to DLB patients with two or more core symptoms recorded (see Table 2). The AD2CS group was younger at referral and diagnosis than patients with dementia in AD with fewer than two DLB core symptoms but older than patients with DLB with two or more core symptoms. While the AD2CS group had a lower proportion of females than the remainder of the AD cohort, it was still more female-dominant than the DLB group. No significant differences in ethnicity were observed between groups. A higher proportion of patients with DLB with two or more core symptoms was married or cohabiting compared to the AD groups.

**Table 2:** Demographic characteristics and core symptoms comparing those with Alzheimer’s disease with 2+ core symptoms to those with Alzheimer’s and one or no core symptom, and to DLB with 2+ core symptoms

	AD with <2 DLB core symptoms (*n* = 11,149)	AD with 2+ core symptoms (AD2CS, *n* = 2563)	DLB with 2+ core symptoms (*n* = 513)	*p*-value (AD with 2+ core symptoms vs <2 core symptoms)	*p*-value (AD with 2+ core symptoms vs DLB with 2+ core symptoms)
Sociodemographic characteristics					
Age at first referral in years (mean, SD)	81.2 (8.1)	77.9 (9.6)	76.8 (10.1)	<0.001	0.013
Age at first dementia diagnosis in years (mean, SD)	82.3 (7.7)	80.2 (8.6)	78.7 (9.0)	<0.001	<0.001
Female gender (%)	64.6%	61.4%	48.5%	0.002	<0.001
Non-White ethnicity (%)	28.2%	26.5%	26.3%	0.089	0.939
Married or cohabiting (%)	34.6%	35.8%	43.1%	0.265	0.003
Core symptoms					
Visual hallucinations (%)	6.7%	60.2%	93.2%	<0.001	<0.001
REM sleep behaviour disorder (%)	4.6%	29.5%	30.6%	<0.001	0.629
Fluctuations (%)	18.4%	81.9%	86.7%	<0.001	0.008
Parkinsonism (%)	11.0%	57.5%	70.4%	<0.001	<0.001

Patients with DLB with at least two core symptoms recorded had a higher mean number of core symptoms (2.8, SD 0.7) than the AD2CS group (2.3, SD 0.5). Among patients with DLB with 2+ core symptoms, visual hallucinations, fluctuations, and parkinsonism were more prevalent than in the AD2CS group, though RBD rates were comparable.

## Discussion

Using machine-learning based NLP applications, we were able to extract clinically relevant symptoms from the electronic health records of over 14,000 patients clinically diagnosed with dementia in Alzheimer’s disease or DLB. Our findings show that nearly one in five patients with dementia in AD exhibited two or more core symptoms of DLB, potentially qualifying them for a diagnosis of probable DLB. This finding underscores the diagnostic challenges in differentiating between AD and DLB, particularly in cases where DLB presents with a symptom profile closely resembling that of AD.

DLB remains significantly underdiagnosed in clinical settings, with many patients first receiving alternative dementia diagnoses, such as dementia in Alzheimer’s disease, before a definitive diagnosis of DLB is made^11^. This has a major impact on patients and their families as DLB has a worse prognosis, including increased mortality and hospitalisation rates^6,12,13^. Previous studies have detected differences in recording of core symptoms of DLB between services^4^, indicating that symptoms like visual hallucinations, cognitive fluctuations, and parkinsonism often go unrecognized or are misinterpreted. The most frequently detected core symptom of DLB in our study was visual hallucinations (83%), which were substantially more prevalent in patients with DLB than in patients with dementia in AD (17%). This finding is consistent with previous studies suggesting that visual hallucinations are a strong diagnostic marker for DLB, as they are much less common in dementia in AD^14^. The high prevalence of visual hallucinations in patients with DLB appears to provide clinicians with a key symptom that can facilitate earlier recognition of the condition.

While visual hallucinations were the most frequently recorded symptom in DLB, fluctuations and parkinsonism also stood out as prominent features of DLB in our cohort. However, fluctuations were also recorded in 30% of AD patients, making it a less specific symptom for DLB diagnosis. The significance of this cannot be understated as fluctuations in cognitively impaired patients have been shown to indicate conversion to DLB^15^, underscoring the complexity of differentiating between AD and DLB based solely on fluctuations. Periods of fluctuations can last from seconds up to several days^16,17^, also depending on subtype diagnosis. Clinicians may overlook fluctuations, mistakenly attributing them to the general cognitive decline associated with AD, rather than recognizing them as a key feature of DLB^18^. The heterogeneity of fluctuations emphasises the need for diverse assessment tools to capture the complexity of fluctuations effectively^17,19^.

Similarly, while parkinsonism was a strong indicator of DLB in our study, with 62% of DLB patients exhibiting bradykinesia or tremor, also 20% of patients diagnosed with dementia in AD exhibited parkinsonism. A previous analysis using NLP to detect Parkinsonian motor symptoms in patients with AD and vascular dementia in the same data source found that parkinsonism was associated with a higher occurrence of neuropsychiatric symptoms at dementia diagnosis, which highlights that parkinsonism might reflect additional Lewy body pathology in those diagnosed with AD or vascular dementia^20^.

REM sleep behaviour disorder was the least commonly recorded symptom in both the DLB and AD groups, with only 27% of DLB patients and 9% of AD patients having RBD noted in their records. RBD, which often manifests as nightmares or bad dreams, is a core symptom of DLB and is associated with early stages of the disease^21^. Our study suggests that RBD is frequently underreported or undiagnosed, possibly due to the symptom’s episodic nature or because it may not be routinely inquired about during clinical assessments. Clinicians may not always ask patients or caregivers about ‘acting out dreams’ or bad dreams or nightmares^22^, which might be particularly difficult in the absence of a bed partner, potentially leading to missed diagnoses. Improved awareness and targeted screening for RBD may help in identifying more cases of DLB, especially in its prodromal stages^21,22^.

When examining the cohort of more than 2500 patients with clinically diagnosed Alzheimer’s disease and 2 + DLB core symptoms, the AD2CS group was similar to those with DLB in respect to age at presentation and age at dementia diagnosis and was also experiencing a longer delay in time to first dementia diagnosis than those with AD and fewer than 2 DLB core symptoms (Average time between first referral and first dementia diagnosis was about one year in patients dementia in AD and < 2 core symptoms, while the average time between first referral and first dementia diagnosis was about two years in the AD2CS group and in patients with DLB an 2+ core symptoms). However, in relation to gender distribution and marital status the AD2CS group was more aligned with patients with AD with fewer than 2 core symptoms. This indicates that only a subgroup of those with AD and two core symptoms will have DLB. However, highlighting this group to clinicians could trigger further examination and re-consideration of the AD diagnosis.

In this context AD and DLB co-pathology need to be considered, as Alzheimer’s co-pathology occurs in half of the cases with Lewy body disease and is associated with earlier mortality, greater cognitive impairment and more rapid cognitive decline^19,23–25^. Analogously, Lewy body co-pathology occurs in at least a quarter of patients with Alzheimer’s disease and has a similar adverse trajectory as AD co-pathology in patients with DLB including more a rapid cognitive decline, earlier mortality, and more widespread cerebral atrophy^26^. From our data it cannot be concluded whether the 19% of patients with AD and two or more core symptoms (AD2CS group) had DLB, mixed AD + DLB, or a mix of other co-pathologies - as up to seven pathologies can be present concurrently in DLB^27^. A study involving an expert assessment of core symptoms in autopsy confirmed cases of AD, DLB and mixed AD + DLB can serve as comparison and provide some insights^28^: While the NLP identified AD2CS group is similar to the autopsy confirmed mixed AD + DLB sample in terms of prevalence of visual hallucinations (AD2CS 60%, autopsy confirmed mixed AD + DLB 50%) and RBD (AD2CS 30%, autopsy confirmed mixed AD + DLB 32%), in relation to Parkinsonism the AD2CS group lies in between with the autopsy confirmed mixed AD + DLB and pure DLB group (autopsy confirmed mixed AD + DLB 45%, AD2CS 58%, autopsy confirmed pure DLB 67%). Fluctuations were more common in the AD2CS group than in any of the autopsy confirmed groups (82% vs 36% in autopsy confirmed mixed AD + DLB or 50% in autopsy confirmed DLB)^28^, which is probably due to the wider definition of fluctuations applied to our sample. Overall, patients with a diagnosis dementia in AD and two DLB core symptoms are probably a mix of pure DLB, mixed AD + DLB, and a mix of other co-pathologies. As both DLB, mixed AD + DLB and other combinations of co-pathologies have an adverse prognosis compared to pure AD^25,26^ a strict distinction might not always be clinically necessary.

A strength of this study is that the application of NLP allowed for an in-depth examination of patient records, revealing patterns of symptoms that may not have been easily detected through reliance on data entered in structured fields. The precision of the NLP algorithms used to detect key symptoms was high, highlighting the utility of NLP in extracting clinically significant data from unstructured text within EHRs. The ability of NLP to process large datasets quickly and efficiently makes it an invaluable tool in clinical settings, especially for identifying underreported symptoms in conditions like DLB.

While the findings of this study are promising, there are some limitations to consider. The retrospective nature of the study and reliance on existing EHRs may have introduced biases, as symptom documentation is dependent on what a clinician asks about in an assessment and what they choose to record; it may thus not fully capture the range of symptoms experienced by patients. Clinicians often only document findings if they consider relevant to clinical care and might for example not document all DLB core symptoms present if the DLB diagnosis is well-established. The study also does not account for inter-clinician variability in symptom recognition and documentation, which could introduce inconsistencies in the data used to train and evaluate the NLP models. In particular, the NLP to capture fluctuations was relatively non-specific capturing any fluctuation in relation to the patient’s presentation. However, this probably reflects the variable definitions of how fluctuations impact a patient’s mental state and functioning, whereby a wide range of manifestations are described from reduced responsiveness to severe changes affecting orientation and behaviour^3,17^. Our finding that fluctuations occurred in 30% of patients with AD is also not dissimilar to what is reported in previous research^29^. In contrast to the other NLP applications, for RBD we were only able to capture symptoms that suggest the possibility of RBD. However, the recurrent dream enactment behaviour which is the hallmark of RBD and usually applied in assessment tools ^22,30^ is probably best captured by ascertaining bad dreams and nightmares in larger scale electronic records.

Future research should aim to refine NLP algorithms to capture a wider range of symptoms associated with DLB and assess the impact of these tools on improving clinical decision-making. As an example, supportive features which are likely to be present in a clinical record, as falls, syncope or signs of autonomic dysfunction^3^, could enhance the sensitivity of combinations of NLP algorithms to detect DLB or even facilitate the development of NLP derived versions of existing scales as the Lewy Body Composite Risk Score^31^. Longitudinal studies tracking patients over time could provide valuable insights into the progression of symptoms in DLB and help establish clearer diagnostic pathways. Moreover, incorporating patient and caregiver reports into EHRs could further enhance the sensitivity of NLP models, especially for symptoms like RBD that are often underreported in clinical settings. An alternative approach that relies less on quality and content of clinical documentation would be the application of convolutional neural models of NLP to free text in electronic patient records. Such models have been shown to distinguish between DLB and AD using data from first consultation up to three months before dementia subtype diagnosis with a precision (positive predictive value) of up to 92%^32^. Difficulties however arose when the NLP models were transferred to another data set and further work is required to develop a more universally applicable and deployable methodology^32^.

Lastly, while the main focus of this analysis was the identification of DLB core symptoms in patients with dementia in AD, in databases of electronic health records diagnoses of vascular, unspecified and other dementias are - with more than 30% of all dementia cases common occurences^33^. Application of NLP to measure the prevalence of core symptoms in these dementia subtypes or all-cause dementia could further enhance case finding of DLB. This approach has for example successfully been applied in a study to identify characteristics associated with progression from very-late onset psychosis to all-cause dementia with ≥2 DLB core symptoms^34^.

In conclusion, the results of this study underscore the potential of NLP in clinical settings. Routine data from EHRs provide valuable opportunities for investigating the course and outcomes of conditions such as dementia over long periods of time, as well as providing observational data on response to interventions. However, research utility is predicated on granularity of data and diagnostic codes are insufficient indicators of dementia phenotypes – particularly important for DLB given its well-recognised under ascertainment. By identifying DLB core symptoms within large research datasets of unstructured EHRs, NLP could facilitate the generation of larger scale research cohorts with a distinct clinical profile. NLP could also be applied to real-time clinical data and support clinicians in recognizing DLB earlier and more accurately. Early diagnosis is critical as it allows for tailored management strategies, including avoiding harmful medications such as antipsychotics, which can exacerbate symptoms in patients with DLB. Moreover, the identification of patients with AD with symptom profiles consistent with DLB provides an opportunity for re-evaluating diagnoses, potentially reducing misdiagnosis rates and improving patient outcomes. Beyond diagnosis, this approach could streamline patient stratification in clinical trials, enabling the inclusion of patients with prodromal or atypical DLB presentations. Integrating NLP into routine practice could enhance clinical decision-making, reduce diagnostic delays, and ultimately improve the quality of care for patients with dementia.

## Methods

### Data source and study cohort

This study utilized the Clinical Record Interactive Search (CRIS) platform, which provides research access to the anonymised electronic health records of patients under South London and Maudsley NHS Foundation Trust (SLaM). SLaM is one of Europe’s largest mental health and dementia care providers, serving the 1.4 million residents of four South London boroughs (Croydon, Lambeth, Lewisham and Southwark). CRIS has research ethical approval as an anonymised database for secondary analysis (Oxford Research Ethics Committee C, reference 23/SC/0257) and data are extracted from structured fields and free text (events, clinical correspondence) through a wide range of natural language processing (NLP) applications^7^. All NLP algorithms plus their performance data are listed on an open-source online catalogue (https://www.maudsleybrc.nihr.ac.uk/facilities/clinical-record-interactive-search-cris/cris-natural-language-processing/)^35^.

From CRIS, we extracted a sample of patients diagnosed with dementia in AD and DLB between January 1, 2008, and December 31, 2021. AD diagnoses were based on structured data fields, specifically F00 WHO ICD-10 codes^36^, while DLB cases were identified using a previously validated NLP tool, which extracts text strings associated with Lewy body dementia^12^.

### Demographics variables

From structured fields within CRIS, we extracted demographic information, including age at first referral to mental health or dementia services, age at initial dementia diagnosis, gender, ethnicity (categorized as White and Non-White), and marital or cohabiting status.

### NLP applications to detect core symptoms of DLB

NLP applications to identify the four DLB core symptoms as per the McKeith criteria - visual hallucinations, cognitive fluctuations, parkinsonism, and REM sleep behaviour disorder (RBD)^3^ - were developed using the GATE (General Architecture for Text Engineering; www.gate.ac.uk) software^37^, an environment for writing applications that can process human language and extract required information as structured data from free-text fields using algorithms developed for this purpose.

Initially instances of a concept in the database are detected using regular expression style matching of keywords. These keywords are defined based on iterative discussions based on what is expected in such a clinical record. Human annotators then label a portion of the sentences containing the concept instances in order to develop a gold standard and training corpus. This allows a machine learning model to be trained, which can ultimately be applied to new, unseen instances to predict the label that should be assigned^38^. As the focus is on ascertaining positive mentions of given entities being present, negation statements (e.g., relevant to this manuscript, ‘not experiencing visual hallucinations’) are therefore not specifically captured as entities (or rated as not present) and are combined with other unwanted text mentions in classification and performance estimation. The performance metrics applied to NLP applications are precision and recall. Precision (positive predictive value) is the proportion of algorithm-derived named entities that are judged to be correct, which is the number of relevant (true positive) entities retrieved as a proportion of the total number of entities retrieved (both irrelevant/false positive and relevant/true positive). Recall (sensitivity) is the proportion of gold standard named entities that are identified by the algorithm, which is the number of relevant (true positive) entities retrieved as a proportion of the total number of relevant (true positive and false negative) entities available in the database. Performance is evaluated by running the NLP application over a corpus of unseen documents, identifying and examining the original clinical document through a linked document identifier, and comparing the results to the manual and NLP coding^7,33,35^.

Specific consideration for the NLP applications to detect four core symptoms of DLB were as follows:

#### Visual hallucinations

Visual hallucinations were detected using an NLP model that identified text strings using the keywords *visual* and hallucinat* in close proximity. Example entities for positive annotations include clinical text such as “responding to visual hallucinations,” “experiencing visual hallucinations,” “history of visual hallucinations,” and “distressed by visual hallucinations”, and for negative annotations “denied any visual hallucinations”, “not responding to visual hallucinations”, and “no (current) visual hallucinations”. Training and test text annotations were sampled from the entirety of the CRIS mental healthcare resource, and not just for people with dementia, which were randomly selected from text strings containing the keyword(s) of interest. Based on a test of 100 clinical documents this application achieved a precision (positive predictive value) of 91% and recall (sensitivity) of 96%.

#### Fluctuations

This machine-learning application captured whether any fluctuations were mentioned in the patient’s record by identifying text strings including the term fluctuat*. This search term was chosen as experience with this specific electronic health records dataset indicates that such terms are preferably used for documentation rather than questions about ‘zoning out’, drowsiness, lethargy or changes in level of functioning, which are suggested in structured DLB assessment toolkits^39^. Fluctuations included needed to refer to any other fluctuations in mental state, as long as they were relevant to the patient’s presentation. Examples of positive annotations include “[patient’s] mood has been fluctuating a lot” or “suicidal thoughts appear to fluctuate”, as well as “fluctuating attention” or “fluctuating cognitive impairment”. Negative annotations included “no evidence of mood fluctuation” or “does not appear to have significant fluctuations in mental state”, and unknown (coded as negative) annotations were “monitoring to see if fluctuations deteriorate”, “his mother’s responsibility fluctuated”, or “is the person’s risk is likely to fluctuate”. As with visual hallucinations, training and test text annotations were sampled from the entirety of the CRIS mental healthcare resource, and not only for people with dementia. Based on a test of 100 clinical documents this application achieved a precision of 87% and recall of 96%.

#### Parkinsonism

We developed two NLP applications to detect the Parkinsonian motor symptoms tremor and bradykinesia. These were developed specifically from text fields in people with a dementia diagnosis and examples of relevant text include “there was evidence of a tremor when writing”, “… with a degree of resting tremor …”, “presence of bradykinesia”, “motor symptoms – moderate bradykinesia L > R”. In 100 documents from people with dementia the application detecting tremor achieved a precision of 83% and recall of 92%. In 100 clinical documents the application to detect bradykinesia achieved a precision of 91% and recall of 84%. Other terms to ascertain Parkinsonian motor symptoms, such as slowness and stiffness were considered, but it was not possible to achieve satisfactory NLP performance metrics in a mental health record database. If tremor, bradykinesia, or both were present, we classified patients as having parkinsonism.

#### REM sleep behaviour disorder (RBD)

The possibility of RBD was estimated if patients had recorded mentions of bad dreams or nightmares in their record. This broad definition was chosen as it captures key presentations of RBD as per screening questionnaires used^40^, while the typical ‘dream enactment’, which usually relies on the patient having a bedpartner, is infrequently described in a mental health record. Capturing sleep disturbances in general is a useful approach to identify DLB as these are considerably more common in patients with DLB compared to patients with AD^41^. As with visual hallucinations and fluctuations, training and test text annotations were sampled from the entirety of the CRIS mental healthcare resource. Positive annotation examples included “had a bad dream last night”, “frequently has bad dreams”, or “unsettled sleep with vivid nightmares”. Both the NLP model for bad dreams and for nightmares achieved a precision of 89% and recall of 100%. A patient was considered to have RBD if bad dreams, nightmares, or both were detected.

### Procedures and statistical analysis

We applied the NLP applications ascertaining DLB core symptoms across the whole anonymised patient electronic health record with no time window restrictions. Analyses were conducted using STATA 18 software (StataCorp, 2023). First, we compared demographics and the prevalence of core symptoms between those with AD and DLB. In a second step we compared those with AD and two or more core symptoms of DLB (AD2CS group) to those with AD with fewer than 2 core symptoms and compared the AD2CS group to patients with a diagnosis of DLB and 2 or more core symptoms recorded. Continuous variables were compared using t-tests and categorical variables using chi-squared tests, with a *p*-value < 0.05 considered significant.

## Data Availability

Data availability statement: All relevant aggregate data are found within the paper. The data used in this work have been obtained from the Clinical Record Interactive Search (CRIS), a system that has been developed for use within the NIHR Mental Health Biomedical Research Centre (BRC) at the South London and Maudsley NHS Foundation Trust (SLaM). It provides authorised researchers with regulated access to anonymised information extracted from SLaM's electronic clinical records system. Individual-level data are restricted in accordance to the strict patient led governance established at South London and The Maudsley NHS Foundation Trust. Data are available for researchers who meet the criteria for access to this restricted data: (1) SLaM employees or (2) those having an honorary contract or letter of access from the trust. For further details, and to obtain an honorary research contract or letter of access, contact the CRIS Administrator at cris.administrator@kcl.ac.uk. Code availability: Code used for this study (STATA do file) is available from the corresponding author on reasonable request.

## Code availability

Code used for this study (STATA do file) is available from the corresponding author on reasonable request.
